# Unifying the Communities of Early‐Onset Glycogen Storage Disease Type IV and Adult Polyglucosan Body Disease Through a Genetic Prevalence Study of *GBE1* ‐Related Disease

**DOI:** 10.1002/jmd2.70080

**Published:** 2026-04-06

**Authors:** Rebecca L. Koch, H. Orhan Akman, Erin Chown, Deberah Goldman, Jeff Levenson, Qing Lu, Lindsay T. Michalovicz Gill, Matthew Morgan, Jennifer L. Orthmann‐Murphy, Natacha T. Pires, Rebecca Reef, Harriet Saxe, Moriel Singer‐Berk, Samantha Baxter

**Affiliations:** ^1^ Division of Medical Genetics, Department of Pediatrics Duke University School of Medicine Durham North Carolina USA; ^2^ Department of Neurology Columbia University Irving Medical Center New York City New York USA; ^3^ Adult Polyglucosan Body Disease Research Foundation Brooklyn New York USA; ^4^ Department of Biostatistics University of Florida Gainesville Florida USA; ^5^ Department of Neurology University of Pennsylvania Philadelphia Pennsylvania USA; ^6^ Program in Medical and Population Genetics Translational Genomics Group Broad Institute of MIT and Harvard Cambridge Massachusetts USA

**Keywords:** adult polyglucosan body disease, Andersen disease, APBD, *GBE1*, glycogen branching enzyme, glycogen storage disease type IV, prevalence

## Abstract

Glycogen storage disease type IV (GSD IV) is an autosomal recessive disorder caused by pathogenic variants in *GBE1*, resulting in deficient glycogen branching enzyme (GBE) activity and formation of abnormal glycogen (“polyglucosan”). GSD IV manifests across a spectrum of clinical dimensions—including hepatic, neurologic, muscular, and cardiac involvement—which vary in severity. The early‐onset forms, historically referred to as Andersen disease, present at different stages ranging from in utero to adolescence. The adult‐onset form, referred to as adult polyglucosan body disease (APBD), typically presents in middle to late adulthood. To date, no epidemiological study of GSD IV has been performed. Understanding the global prevalence of GSD IV is critical to increase disease awareness, improve diagnostic rates, inform therapeutic development, and engage pharmaceutical companies. In collaboration with the Rare Genomes Project at the Broad Institute of MIT and Harvard and the APBD Research Foundation, this study curated variants in *GBE1* and calculated prevalence across nine genetic ancestry groups. The estimated global carrier frequency of GSD IV is 1 in 243 individuals, and the global genetic prevalence is 1 in 235 784 individuals. Based on the 2024 world population, the estimated number of affected individuals with GSD IV is approximately 34 800. These estimates highlight a significant underdiagnosis of GSD IV and underscore the urgent need for increased awareness of this metabolic disorder. This model of collaboration between researchers, patient advocacy organizations, and genetic data sharing programs provides a framework for estimating the prevalence of other rare diseases in the global population.

## Summary

- The estimated global carrier frequency of glycogen storage disease type IV is 1 in 243 individuals.
- The estimated global genetic prevalence of glycogen storage disease type IV is 1 in 235 784 individuals.
- Based on the 2024 world population estimates, there are approximately 34 800 individuals with glycogen storage disease type IV (including the adult‐onset form adult polyglucosan body disease).

## Graphical abstract

Genetic prevalence study of glycogen storage disease type IV. In collaboration with the Rare Genomes Project at the Broad Institute of MIT and Harvard and the APBD Research Foundation, this study queried and curated variants in *GBE1* from ClinVar, HGMD, and gnomAD to calculate the genetic prevalence of glycogen storage disease type IV (GSD IV). The estimated global carrier frequency of GSD IV is 1 in 243 individuals, and the global genetic prevalence is 1 in 235 784 individuals. Based on the 2024 world population, the estimated number of affected individuals with GSD IV is approximately 34 800. These estimates include all forms of glycogen storage disease type IV, including the adult‐onset form adult polyglucosan body disease (APBD). Created in BioRender. Koch, R. (2025) https://BioRender.com/j0sg30n.

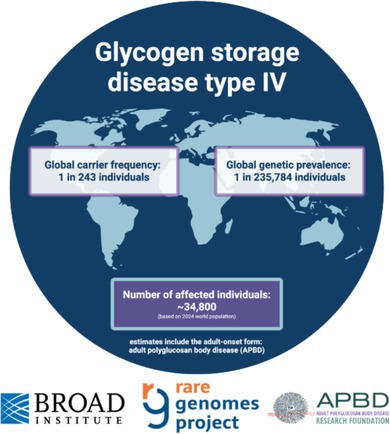

## 1 Introduction

Glycogen storage disease type IV (GSD IV) is caused by biallelic pathogenic variants in *GBE1* (HGNC: 4180) which results in reduced activity of glycogen branching enzyme (GBE, EC 2.4.1.18). As a result, glycogen synthesis is impaired and poorly branched glycogen‐like material (“polyglucosan”) accumulates in cells, ultimately aggregating into polyglucosan bodies that disrupt cellular function [1]. Phenotypically, GSD IV is incredibly heterogeneous and is conceptualized as a multidimensional clinical continuum with hepatic, neurologic, muscular, and cardiac involvement occurring to varying degrees of severity [2]. The early‐onset forms—initially referred to as Andersen disease and later classified as GSD IV (OMIM #232500) – include presentations in utero, during infancy, childhood, and adolescence. The adult‐onset form, referred to as adult polyglucosan body disease (APBD, OMIM #263570), is a neurodegenerative disease with typical presentations in middle to late adulthood. Numerous pathogenic variants in *GBE1* have been reported with varying impacts on residual GBE activity levels, underscoring the disease heterogeneity [1]. However, genotype–phenotype relationships remain poorly understood.

GSD IV, including the early‐onset and APBD forms, is classified as an ultra‐rare autosomal recessive disease and has been reported in various ethnic groups [2, 3]. Prevalence of a disease is an important factor in decision‐making by researchers, pharmaceutical companies, and policymakers to determine goals and resource allocation toward the disease, yet the prevalence of the vast majority of rare diseases is unknown. Increased knowledge about the prevalence of a disease, both globally and in specific genetic ancestry groups, is important to patient advocacy and research organizations that aim to extend outreach and support to build a network that engages as many affected individuals and families as possible.

To date, a formal epidemiological study of all GSD IV phenotypes has not been performed. A general prevalence estimate of 1 in 600 000 to 800 000 individuals was proposed [4]. Additional prevalence and carrier frequency studies have been focused on the APBD form. In 2012, the first epidemiological study of the *GBE1* c.986A>C (p.Tyr329Ser) variant was published [5]. This variant causes APBD when homozygous or compound heterozygous with a deep intronic variant [6]. It occurs at a higher frequency in the Ashkenazi Jewish ancestry group, with this study estimating the carrier frequency of APBD to be approximately 1 in 35 individuals of Ashkenazi Jewish ancestry [5]. Then, based on next‐generation carrier screening performed in 2016, Sema4 expanded on this and included the c.986A>C (p.Tyr329Ser) variant along with other *GBE1* variants observed in patients with APBD; they estimated the carrier frequency for APBD in individuals of Ashkenazi Jewish ancestry to be 1 in 48 [7, 8]. Then, a follow‐up investigation was performed in 2020 to calculate the prevalence of APBD, estimating that there are 3400 to 6400 individuals of Ashkenazi Jewish ancestry over the age of 50 years with APBD in the United States [8]. Notably, this report also highlighted the high misdiagnosis rate of APBD with multiple sclerosis in the United States.

Despite the importance of these initial carrier frequency and prevalence studies on APBD, they are inherently limited in their application to the broader GSD IV community. The aforementioned studies did not include prevalence estimates of ethnicities other than Ashkenazi Jewish ancestry and were restricted to those with the APBD form rather than all patients with GSD IV. This study expands on previous efforts and produces estimates of the global carrier frequency and genetic prevalence of all GSD IV forms, including the early‐onset and APBD forms. Appreciating the unmet medical need in the GSD IV community—which drives research into disease diagnosis, management, and treatment—requires that we develop a clearer understanding of the global prevalence of this disease.

## 2 Methods

### 2.1 Nomenclature

Consistent with the clinical practice guidelines for GSD IV [1], we herein considered GSD IV to be a continuum of disease, varying by age of onset and organ involvement. In recognition of the APBD form having a relatively distinct collection of neurological symptoms and typical age of onset in adulthood, we refer to those with onset in childhood or adolescence as “early‐onset GSD IV” and those with adult‐onset GSD IV as “adult polyglucosan body disease” (APBD).

### 2.2 Study Design

In collaboration with the Rare Genomes Project at the Broad Institute of MIT and Harvard and the APBD Research Foundation, this study was conducted to estimate the global genetic prevalence of all known disease‐causing variants in *GBE1* (GRCh37, ENST00000429644.2). A comprehensive list of suspected pathogenic variants in *GBE1* that were present in Genome Aggregation Database (gnomAD) [9, 10] was assembled for variant curation according to American College of Medical Genetics and Genomics/Association for Molecular Pathology (ACMG/AMP) Guidelines for Sequence Variant Interpretation [11, 12, 13]. Sources for genetic variants included ClinVar (pathogenic/likely pathogenic/conflicting with at least one source saying pathogenic/likely pathogenic) [14], the Human Gene Mutation Database (HGMD; disease‐causing mutation) [15], as well as any other high‐confidence predicted loss‐of‐function (pLoF) variants in gnomAD reported as of November 20, 2024. The duration of this project spanned two versions of gnomAD (v2 and v4). All variants that met our criteria in v2 received a full ACMG/AMP and pLoF curation; however, due to the large number of new variants introduced in v4 (*n* = 200), we designed a triaged approach to review all new v4 variants. Any variant with an allele count (AC) >/= 15 (allele frequency (AF) > 0.00001) received a full ACMG/AMP curation, and any pLoF variant with AC > 1 received LoF curation (Supporting Information Table S1) [9]; variants with an AC < 15 received an abbreviated curation (e.g., literature search). All variants which met our criteria in v2 but were initially classified as variants of uncertain significance (VUSs) were re‐curated to ensure there was no new evidence that would change the classification.

Variants classified as VUSs from ClinVar and other databases were not included in this analysis as including these would inaccurately inflate the estimated prevalence of disease due to the potential of them being classified as benign when more evidence is gathered over time. In addition, the variants that were originally listed as pathogenic, likely pathogenic, or conflicting in ClinVar or disease‐causing mutation in HGMD, but were classified as VUSs after curation, are included in this analysis as VUSs. Full details on the variant list creation and curation are found in the Supporting Information. After curation was performed, the AF were collected from gnomAD v4.1. Variants not present in gnomAD were not included in the calculations since their gnomAD AF would equal zero and therefore would not impact the estimates. The aggregate carrier frequency was estimated on a global level by distinct genetic ancestry groups. The Hardy–Weinberg principle was then applied where q = variant AF, *Σ*q = aggregate AF, 2*Σ*q = cumulative carrier frequency, (*Σ*q)^2^ = genetic prevalence. “Conservative” prevalence estimates include only pathogenic and likely pathogenic variants (excluding VUSs), and “relaxed” prevalence estimates include pathogenic variants, likely pathogenic variants, and VUSs (Supporting Information Table S2). The curated annotations of predicted protein‐truncating variants were released to the public through the gnomAD browser, and the variant curations were submitted to ClinVar.

## 3 Results

**FIGURE 1:**
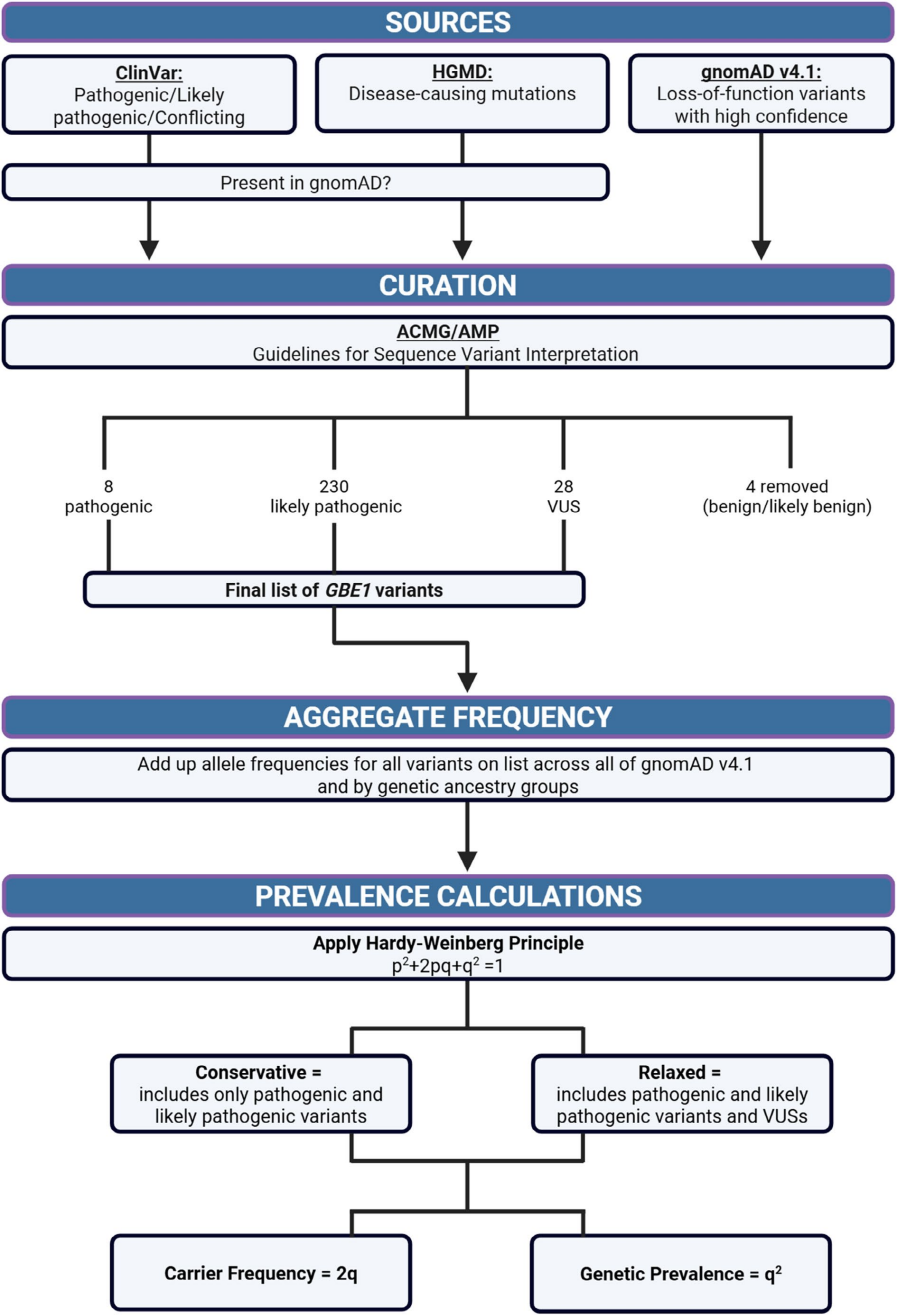
Overview of the genetic prevalence study of GSD IV conducted by the Rare Genomes Project at the Broad Institute of MIT and Harvard in collaboration with the APBD Research Foundation. Created in BioRender. Koch, R. (2025) https://BioRender.com/7uj18lq.

Variant databases ClinVar, HGMD, and gnomAD were queried to compile a list of variants thought to be associated with GSD IV, including early‐onset and APBD phenotypes, in order to estimate the genetic prevalence for this recessive disease (Figure 1). Each variant was standardized using the GRCh38 genome build and the canonical transcript NM_000158.4. After aggregating the list of variants and accounting for duplicates (Supporting Information Figure S1), 104 variants met our criteria for full ACMG/AMP and/or LoF curation, and 166 received an abbreviated curation. Ultimately, 4 variants were removed from consideration because they were classified as benign/likely benign. In the end, a total of 266 variants in *GBE1* were included in the carrier frequency and prevalence calculations: 8 pathogenic, 230 likely pathogenic, and 28 VUSs. Of note, the 28 VUSs do not include all VUSs from ClinVar, but rather the pathogenic/likely pathogenic variants that were downgraded to a VUS after a full ACMG/AMP curation.

**FIGURE 2:**
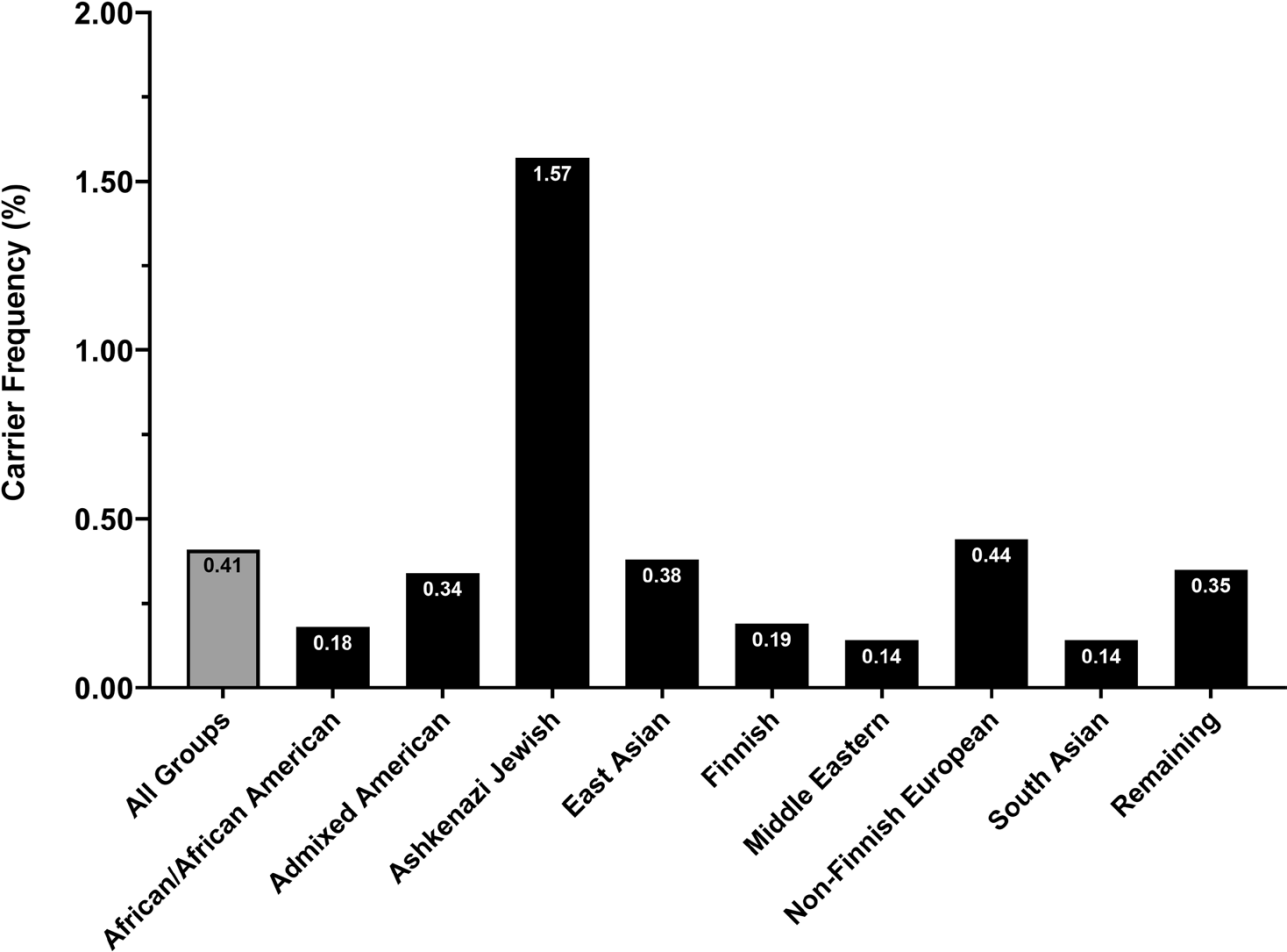
Group‐specific carrier frequencies of GSD IV. The conservative carrier frequencies were calculated using pathogenic/likely pathogenic variants in *GBE1*. The carrier frequency (represented as a percentage of people) was rounded to the nearest hundredths value for individuals in all genetic ancestry groups (All Groups, gray) as well as by specific genetic ancestry groups (black).

Based on calculations with only pathogenic and likely pathogenic variants in *GBE1*, the estimated conservative global carrier frequency of all GSD IV phenotypes (i.e., early‐onset and APBD) is 1 in 243 individuals, and the conservative global genetic prevalence of GSD IV is approximately 1 in 235 800 individuals (Table 1 and Figure 2, Graphical Abstract). Based on the 2024 world population (8.2 billion), the conservative estimated number of affected individuals with GSD IV is approximately 34 800. The carrier frequency varied amongst genetic ancestry groups. As expected, there was a significantly higher conservative carrier frequency in the Ashkenazi Jewish group (1 in 64 individuals). The next most frequent carrier was the non‐Finnish European group (1 in 227 individuals), followed by the East Asian group (1 in 265 individuals) and the Admixed American group (1 in 293 individuals). Based on the 2024 United States and European population estimates of 336 and 745 million, respectively, and the overall (all groups) carrier frequency, there are an estimated 1452 individuals with GSD IV in the United States compared to an estimated 3160 individuals with GSD IV in Europe. When specifying these estimates based on non‐Finnish European carrier frequency, there are an estimated 3625 affected individuals in Europe.

**TABLE 1:** Global carrier frequency (2q) and genetic prevalence (q^2^) of GSD IV by genetic ancestry group.

	Individuals	Pathogenic/likely pathogenic variants	Carrier frequency (2q)	Genetic prevalence (q^2^)
All groups	807 162	238	1/243	1/235776
African/African American	30 019	32	1/564	1/1270243
Admixed American	37 545	32	1/293	1/342705
Ashkenazi Jewish	14 804	5	1/64	1/16258
East Asian	22 448	20	1/265	1/281930
Finnish	32 026	11	1/531	1/1127547
Middle Eastern	3031	4	1/733	1/2148714
Non‐Finnish European	590 031	170	1/227	1/205539
South Asian	45 546	31	1/738	1/2180633
Remaining	31 256	32	1/289	1/335134

*Note:* Calculations are based on conservative estimates which include pathogenic and likely pathogenic variants and exclude variants of uncertain significance in *GBE1*. The number of individuals indicates the representation of individuals in gnomAD v4.1. Similarly, the number of pathogenic/likely pathogenic variants indicates how many pathogenic/likely pathogenic variants in *GBE1* (based on ACMG/AMP Guidelines for Sequence Variant Interpretation) were observed in each genetic ancestry group. The aggregate carrier frequency and genetic prevalence was estimated for each genetic ancestry group based on the Hardy–Weinberg principle. Additional details, including allele counts and allele frequencies for each variant by genetic ancestry group, are available through the Genetic Prevalence Estimator (GeniE) tool at https://broad.io/GeniE_GBE1.

Among the 238 pathogenic and likely pathogenic variants in *GBE1*, the variants that occurred in the highest frequency across all genetic ancestry groups were c.691+2T>C (p.?, 0.20% carrier frequency) and c.986A>C (p.Tyr329Ser; 0.03% carrier frequency). The c.691+2T>C variant was observed across all genetic ancestry groups except within the Admixed American, East Asian, and South Asian groups, with the highest incidence in the Non‐Finnish European group (0.25% carrier frequency). The c.986A>C (p.Tyr329Ser) variant was most commonly observed in the Ashkenazi Jewish group (1.41% carrier frequency), and it was also observed—albeit more rarely—in the Non‐Finnish European and the Remaining groups (0.006% and 0.10% carrier frequency, respectively). Though gene deletions and deep intronic variants have been reported in *GBE1* [16], whole genome sequencing data was only available from a select number of individuals in gnomAD v4 (76 215/807162 total individuals [17]), and we were therefore not able to calculate their carrier frequencies.

The relaxed (i.e., including VUSs in *GBE1*) carrier frequency and genetic prevalence results are included in Supporting Information Table S3 and Figure S2.

## 4 Discussion

Understanding the carrier frequency and genetic prevalence of GSD IV is critical to improving diagnoses, supporting outreach to carriers and individuals with GSD IV, informing therapeutic development, and engaging with pharmaceutical companies.

This study builds on previous work by offering global prevalence estimates for all known pathogenic/likely pathogenic *GBE1* variants, capturing the full spectrum of GSD IV, from early‐onset to adult‐onset APBD. Pooling variants from ClinVar, HGMD, and gnomAD and classifying them according to the ACMG/AMP criteria, we calculated the genetic prevalence of GSD IV to be approximately 1 in 235 800 individuals. This estimate includes individuals who are both symptomatic and presymptomatic. These genetic prevalence estimates do not account for the possibility of certain allele combinations leading to early miscarriage or stillbirth; therefore, those impacted pregnancies would also be accounted for in these estimates. Based on the 2024 world population, there is an estimated 34 800 affected individuals with GSD IV. The large discrepancy between these calculations and the number of cases reported in the literature may be due to a high rate of misdiagnosis and delayed diagnosis. These persistent diagnostic challenges were highlighted during the APBD Patient‐Led Listening Session with the FDA in October 2021 [18]; testimonies at the session revealed that patients often experience a constellation of symptoms for years, with reported diagnostic journeys that far exceed the cited average of 6.8 years [19], frequently misattributed to more common conditions (e.g., multiple sclerosis). Contributing factors were highlighted in the recent clinical practice guidelines for GSD IV and may include misdiagnoses, undergoing genetic testing with a targeted gene panel that excludes *GBE1* or with testing that does not cover deep intronic variants or large deletions, and failure to pursue further evaluation after negative or inconclusive results [1]. Compounding these issues is a broader lack of awareness of GSD IV and its phenotypic continuum within the healthcare community. This study reinforces the urgent need to confront these barriers through improved diagnostic strategies and continued advocacy for research and treatment for the GSD IV community.

The number of pathogenic/likely pathogenic *GBE1* variants and broad phenotypic heterogeneity of GSD IV depicts a complex relationship between genotype and phenotype. Interestingly, there have been reports of individuals with early‐onset GSD IV or APBD with the same *GBE1* genetic variants [20, 21], patients with traditional features of hepatic early‐onset GSD IV who later developed symptoms consistent with APBD in adulthood [21, 22], as well as patients who developed symptoms consistent with APBD but at an earlier age [23, 24]. Therefore, recent studies have suggested that GSD IV is a phenotypic continuum with a spectrum of involvement from neurologic, muscular, hepatic, and cardiac systems with varying degrees of severity [2]. Given this genotypic overlap, we were unable to calculate the genetic prevalence of the early‐onset vs. APBD forms. However, it is well documented that individuals who are homozygous for the *GBE1* c.986A>C (p.Tyr329Ser) variant manifest the APBD form, and that this variant occurs at a higher frequency in the Ashkenazi Jewish ancestry group [5]. Based on our estimated carrier frequency in the Ashkenazi Jewish ancestry group, 1/20069 individuals of Ashkenazi Jewish genetic ancestry are homozygous for the c.986A>C variant. Assuming there are an estimated 11 million Ashkenazi Jews in the world, that equates to ~550 Ashkenazi Jews who harbor the c.986A>C variant in homozygosity and are expected to have APBD. Yet, there are several other genotypes associated with the APBD form [1, 3, 25], as well as recognition of individuals who present with early‐onset disease and then develop symptoms consistent with APBD in adulthood [20, 21], making it difficult to define “APBD” in this context. Therefore, our estimate of ~550 individuals is likely a small proportion of all affected individuals with APBD.

Early‐onset GSD IV cases have been reported in Europe, the Middle East, South Asia, and North and South America, among others [2, 20]. Based on our genetic prevalence estimates, there are more affected individuals in Europe (3160 individuals) than in the United States (1452 individuals). In recent years, the APBD Research Foundation has taken steps to strengthen engagement within the European community [26]. This outreach is an essential step towards closing the gap between the global prevalence estimates and confirmed diagnoses, especially as improved awareness and diagnostic access are key to identifying misdiagnosed and undiagnosed individuals. Interestingly, despite previous reports of GSD IV in India and other South Asian populations [20], the carrier frequency was the lowest in this group at 1 in 738 individuals; this is likely explained by a reduced representation of these populations in gnomAD v4 [27], and the carrier frequency may actually be higher in certain ancestry groups, such as the Indian population. In addition, while the relaxed estimates (i.e., incorporating including pathogenic and likely pathogenic variants along with VUSs) were higher in all genetic ancestry groups compared to conservative estimates (i.e., incorporating only pathogenic and likely pathogenic variants), the estimates were strikingly different in the Middle Eastern genetic ancestry group. This difference was primarily driven by the inclusion of c.986A>G (p.Tyr329Cys) in the relaxed calculation. Should this variant be reclassified as pathogenic or likely pathogenic in the future, the carrier frequency and genetic prevalence estimates would be expected to increase in the Middle Eastern genetic ancestry group. However, it is important to remember that the Middle Eastern population in gnomAD is only 3031 individuals (0.38% of gnomAD v4) [17], and smaller sample sizes are more susceptible to ascertainment bias and inflated AFs due to the smaller overall allele count.

APBD has also been reported in several populations [24, 28, 29, 30, 31, 32, 33, 34, 35, 36, 37, 38], but is known to be more common in individuals of Ashkenazi Jewish genetic ancestry [25]. According to our calculations, the carrier frequency of GSD IV in Ashkenazi Jews is 1 in 64 individuals, slightly lower than previously reported estimates (1 in 35–48 individuals) [5, 7, 8]. It is important to note that our calculations include both early‐onset GSD IV and APBD, whereas the previous estimates were specifically calculated for individuals with the APBD phenotype based on *GBE1* genotype, age of onset, or both. Moreover, our estimates were not able to include the c.2053‐3358_2053‐3350delins deep intronic variant associated with APBD [6]. Therefore, the previously reported estimates likely hold merit when estimating prevalence and carrier frequency specifically for “classic” APBD (i.e., homozygous for the c.986A>C (p.Tyr329Ser) variant in *GBE1* or compound heterozygous with the c.986A>C (p.Tyr329Ser) and the c.2053‐3358_2053‐3350delins variants [6]).

As emerging evidence suggests that certain variants in *GBE1* can lead to both early‐onset GSD IV and APBD, there may be a growing rationale for broader inclusion of *GBE1* in reproductive/carrier screening panels for populations with higher carrier frequencies. The carrier frequency in the Ashkenazi Jewish genetic ancestry group (1 in 64) is comparable to that of other conditions routinely screened for in this population, falling somewhere between Canavan disease (1 in 40–82 individuals [39]) and Bloom syndrome (1 in 111–157 individuals [40]). Additionally, there is a known prevalence of GSD IV in the Mennonite communities [41] which was not captured in this dataset. Ensuring that *GBE1* is included on carrier screening panels—even for those not of Ashkenazi and Mennonite genetic ancestry—may be a helpful solution to the pervasive problem of misdiagnosis and may help close the gap between predicted and currently identified affected individuals. However, the ethical implications of screening for a disease with an unpredictable age of onset—from childhood to adulthood—as well as the lack of disease‐modifying therapies warrant further exploration and discussion, as most of these panels test for pediatric‐onset conditions. A similar concern has been raised for carrier screening panels that test for Gaucher disease given carriers of pathogenic variants in *GBA1* (HGNC:4177) are at a higher risk of developing Parkinson's disease compared to the general population [42].

**FIGURE 3:**
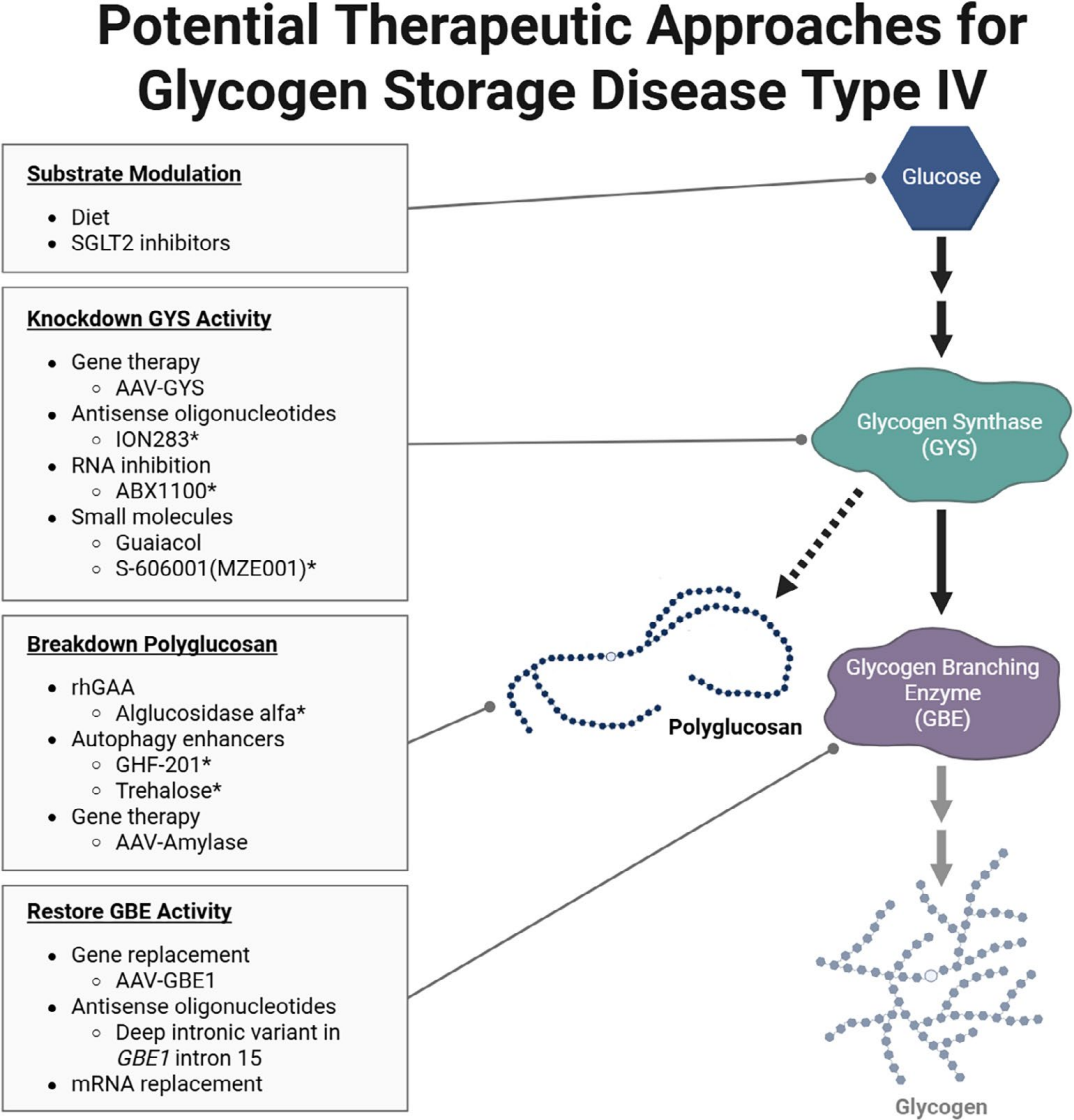
Potential therapeutic approaches for GSD IV. There is a range of therapeutic approaches under investigation for GSD IV (glycogen branching enzyme [GBE] deficiency) and allied GSDs. Many of these approaches target different aspects of the glycogen synthesis and polyglucosan breakdown. These approaches include substrate modulation, reducing glycogen synthase (GYS) activity (GYS1 or GYS2 isoforms), breaking down polyglucosan inclusions, and restoring GBE activity. This list is not exhaustive, rather it offers potential therapeutic opportunities that may or may not have been investigated. Not featured is transplantation of the liver or heart in cases of liver and heart failure, respectively. *indicates that this approach has been investigated in patients with a related GSD (i.e., Pompe disease or Lafora disease) or healthy volunteers as of January 2026. Additional abbreviations: AAV, adeno‐associated virus; DNA, deoxyribonucleic acid; mRNA, messenger ribonucleic acid; rhGAA, recombinant human acid alpha‐glucosidase; RNA, ribonucleic acid. Created in BioRender. Koch, R. (2026) https://BioRender.com/qv9q5qn.

Prevalence estimates can inform the design of clinical trials, support regulatory planning, and guide investment in therapeutic development, ensuring that emerging treatments reach this patient population. To date, there are no treatment options for GSD IV, and care remains focused on managing symptoms. Organ transplantation of the liver or heart can be lifesaving but will not address the extra‐hepatic and extra‐cardiac disease manifestations, respectively [1]. A range of promising therapeutic approaches are under investigation (Figure 3). In theory, adjustment of carbohydrate intake [1, 43] or use of sodium‐glucose cotransporter 2 (SGLT2) inhibitors could reduce the glucose substrate used to form polyglucosan; the latter has been proposed for therapeutic use in Lafora disease—a related GSD with polyglucosan accumulation [44, 45]. Researchers have also investigated adeno‐associated virus (AAV)‐mediated downregulation of GYS1 to reduce glycogen synthase (GYS) activity in the central nervous system, muscle, and other tissues in GSD IV [46, 47]. There are additional GYS1‐targeting therapies being investigated for other GSDs that could theoretically treat GSD IV, such as intrathecally administered antisense oligonucleotide (ASO) therapy targeting GYS1 for Lafora disease (ION283, NCT06609889), RNA interference‐mediated GYS1 reduction for Pompe disease (ABX1100, NCT06109948) [48], and small molecule GYS1 inhibitor for Pompe disease (S‐606001, formerly MZE001, NCT07123155) [49]. In addition, gene replacement therapy candidates currently under investigation aim to restore GBE activity [50]. Meanwhile, n‐Lorem is pursuing a precision ASO therapy for a small group of APBD patients with a deep intronic variant in *GBE1* [6, 51]. Using mRNA therapy to restore GBE activity levels has not been investigated in GSD IV but is of theoretical benefit. Researchers are also investigating treatments for GSD IV that work through alternative mechanisms. This includes small molecule autophagy enhancer GHF‐201, which is being assessed in healthy adult volunteers (EUCT 2024–519 813–74–00) [52], supplementation of guaiacol (a GYS inhibitor) [53], alglucosidase alfa to enhance lysosomal polyglucosan degradation [54], and AAV‐mediated delivery of cross‐correction‐enabled amylase [55]. Lastly, compounds tested in other related GSDs, such as autophagy inducer trehalose for Lafora disease [56, 57], may be worth investigating in GSD IV. It is important to note that this list is not exhaustive and with the improvements in technologies like machine learning models, there may be opportunities to repurpose drugs for the treatment of GSD IV [58]. As the therapeutic pipeline for GSD IV continues to grow, genetic prevalence studies become increasingly critical to aid in identifying how many individuals are affected by the disease. Moreover, additional research to expand upon this prevalence data—such as understanding the prevalence of specific variants and/or genotype–phenotype relationships—could help identify specific populations that might be most responsive to a particular therapy.

As the therapeutic pipeline for GSD IV continues to grow, genetic prevalence studies become increasingly critical to aid in identifying how many individuals are affected by the disease. Importantly, prevalence estimates can inform the design of clinical trials, support regulatory planning, and guide investment in therapeutic development, ensuring that emerging treatments reach the patient populations for whom there currently are no approved treatment options.

This study estimated carrier frequency and disease prevalence for recessive conditions using curated variants in *GBE1*. This methodology has been used for genetic prevalence studies of several genetic diseases [59], such as for phospholipase A2 group VI (PLA2G6)‐associated neurodegeneration which was done by the Rare Genomes Project at the Broad Institute of MIT and Harvard [60]. There are many factors that go into calculating the estimated carrier frequencies and genetic disease prevalence, such as available data in ClinVar (database of classified variants) and gnomAD (reference population database), as well as curation guidelines. It is important to consider that these calculations are estimates and include inherent limitations. While gnomAD is the world's largest human reference database, it is not representative of the entire global population. Some genetic ancestry groups are either underrepresented or still missing from gnomAD and more diversity of reference data is needed. The estimates reported in this study are influenced by the genetic ancestral makeup of gnomAD, which is heavily composed of individuals of European genetic ancestry [61]. While future releases of gnomAD—which will include adding National Institutes of Health All Of Us data [62] along with efforts to federate other worldwide databases [63]—are expected to increase representation of diverse genetic ancestries, this limitation should be considered when interpreting these results. Given the relatively smaller sample size of some genetic ancestry groups in gnomAD, caution should be used when making conclusions about group‐specific trends. Moreover, there may have been some reported pathogenic and likely pathogenic variants that were not present in gnomAD and therefore could not be included in this analysis. Future versions of gnomAD are expected to include more samples with whole genome sequencing which will improve calculation estimates of variants that are not detected with whole exome sequencing, such as deep intronic variants and large deletions. In addition, the calculations used in this study do not take into account the increased rates of consanguinity in some cultures which increase the prevalence of recessive conditions in these regions. Lastly, we excluded VUSs—which were often missense variants—from our conservative estimates, yet with GBE testing to biochemically support the diagnosis as well as improved understanding of the effect of these missense variants on the protein function, it is expected that some VUSs may be able to be reclassified as pathogenic or likely pathogenic. It is important to note that the prevalence numbers reported here may change in the future as technologies evolve, AFs are refined with more uniform data sharing policies, and disease natural history data is published.

## 5 Conclusion

Our genetic prevalence study of GSD IV estimated that there are approximately 34 800 individuals affected by GSD IV globally, with an overall carrier rate of 1 in 243 individuals. Though the APBD form remains prevalent in the Ashkenazi Jewish group, this study highlights the genetic prevalence of GSD IV across a variety of ancestry groups. This comprehensive genetic prevalence study paves the way for future clinical trial design as researchers assess the potential impact of therapies for GSD IV on the global population. In addition, having a more accurate prevalence estimate is essential for reducing the rate of misdiagnosis and diagnostic delay that affected patients often report experiencing. This model of collaboration between researchers, patient advocacy organizations, and genetic data sharing programs provides a framework for estimating the prevalence of other rare diseases in the global population.

## Author Contributions

**Rebecca L. Koch:** investigation, formal analysis, writing – original draft, writing – review and editing; **H. Orhan Akman:** writing – review and editing; **Erin Chown:** writing – review and editing; **Deberah Goldman:** writing – review and editing; **Jeff Levenson:** writing – review and editing; **Qing Lu:** writing – review and editing; **Lindsay T. Michalovicz Gill:** writing – review and editing; **Matthew Morgan:** writing – review and editing; **Jennifer L. Orthmann‐Murphy:** writing – review and editing; **Natacha T. Pires:** supervision, writing – review and editing; **Rebecca Reef:** writing – review and editing; **Harriet Saxe:** writing – review and editing; **Moriel Singer‐Berk:** methodology, formal analysis; **Samantha Baxter:** funding acquisition, investigation, methodology, formal analysis, supervision, writing – review and editing.

## Funding

This work was supported by the Chan Zuckerberg Initiative Donor‐Advised Fund at the Silicon Valley Community Foundation 2020‐224274 and 2022‐316726 (https://doi.org/10.37921/236582yuakxy) (funder DOI 10.13039/100014989).

## Ethics Statement

The authors have nothing to report.

## Consent

The authors have nothing to report.

## Conflicts of Interest

Rebecca L. Koch has received grant/funding support from Alnylam Pharmaceuticals. Rebecca L. Koch and H. Orhan Akman have received grant/funding support from the Keith B. Hayes Foundation. Jennifer Orthmann‐Murphy is a consultant for Vigil Neuroscience and Savanna Bio as well as a site PI for Vigil Neuroscience clinical trials. Samantha Baxter is a paid consultant for Pharming Group N.V. The Adult Polyglucosan Body Disease Research Foundation is a shareholder of Golden Heart Flower Ltd., holds a revenue sharing agreement with Columbia University for treatment development of guaiacol, and has received grant/funding support from the Chan Zuckerberg Initiative. All other authors declare no conflicts of interest.

## Supporting information


**Table S1:** Loss‐of‐Function (LoF) curation and error mode descriptions.
**Table S2:** Frequency calculation descriptions.
**Figure S1:** Contribution of *GBE1* variants from each database.
**Table S3:** Global carrier frequency (2q) and genetic prevalence (q2) of GSD IV by genetic ancestry group.
**Figure S2:** Group‐specific carrier frequencies of GSD IV.

## Data Availability

All estimated genetic prevalence reports are publicly available through the Genetic Prevalence Estimator (GeniE) tool. Specifically, the conservative estimates (i.e., only includes pathogenic and likely pathogenic variants in *GBE1*) are available at https://broad.io/GeniE_GBE1 and the relaxed estimates (i.e., includes pathogenic and likely pathogenic variants as well as variants of uncertain significance in *GBE1*) are available at https://broad.io/GeniE_GBE1_relaxed.

## References

[1] R. L. Koch , C. Soler‐Alfonso , B. T. Kiely , et al., “Diagnosis and Management of Glycogen Storage Disease Type IV, Including Adult Polyglucosan Body Disease: A Clinical Practice Resource,” Molecular Genetics and Metabolism 138, no. 3 (2023): 107525. 36796138 10.1016/j.ymgme.2023.107525

[2] B. T. Kiely , R. L. Koch , L. Flores , D. Burner , S. Kaplan , and P. S. Kishnani , “A Novel Approach to Characterize Phenotypic Variation in GSD IV: Reconceptualizing the Clinical Continuum,” Frontiers in Genetics 13 (2022): 992406. 36176296 10.3389/fgene.2022.992406 PMC9513518

[3] F. Mochel , R. Schiffmann , M. E. Steenweg , et al., “Adult Polyglucosan Body Disease: Natural History and Key Magnetic Resonance Imaging Findings,” Annals of Neurology 72, no. 3 (2012): 433–441. 23034915 10.1002/ana.23598 PMC4329926

[4] P. L. Magoulas and A. W. El‐Hattab , “Glycogen Storage Disease Type IV,” in GeneReviews, ed. M. P. Adam (University of Washington, 1993). 23285490

[5] A. Hussain , J. Armistead , L. Gushulak , et al., “The Adult Polyglucosan Body Disease Mutation GBE1 c.1076A>C Occurs at High Frequency in Persons of Ashkenazi Jewish Background,” Biochemical and Biophysical Research Communications 426, no. 2 (2012): 286–288. 22943850 10.1016/j.bbrc.2012.08.089

[6] H. O. Akman , O. Kakhlon , J. Coku , et al., “Deep Intronic GBE1 Mutation in Manifesting Heterozygous Patients With Adult Polyglucosan Body Disease,” JAMA Neurology 72, no. 4 (2015): 441–445. 25665141 10.1001/jamaneurol.2014.4496

[7] G. Akler , A. H. Birch , N. Schreiber‐Agus , et al., “Lessons Learned From Expanded Reproductive Carrier Screening in Self‐Reported Ashkenazi, Sephardi, and Mizrahi Jewish Patients,” Molecular Genetics & Genomic Medicine 8, no. 2 (2020): e1053. 31880409 10.1002/mgg3.1053 PMC7005669

[8] L. R. Schwartz , Q. Lu , R. Liu , et al., “Estimating the Prevalence of the Adult Polyglucosan Body Disease at the Gene Level for Ashkenazi Jews in the United States,” American Journal of Rare Disorders: Diagnosis & Therapy 3, no. 1 (2020): 4–8.

[9] M. Singer‐Berk , S. Gudmundsson , S. Baxter , et al., “Advanced Variant Classification Framework Reduces the False Positive Rate of Predicted Loss‐of‐Function Variants in Population Sequencing Data,” American Journal of Human Genetics 110, no. 9 (2023): 1496–1508. 37633279 10.1016/j.ajhg.2023.08.005 PMC10502856

[10] S. Chen , L. C. Francioli , J. K. Goodrich , et al., “A Genomic Mutational Constraint Map Using Variation in 76,156 Human Genomes,” Nature 625, no. 7993 (2024): 92–100. 38057664 10.1038/s41586-023-06045-0 PMC11629659

[11] A. N. Abou Tayoun , T. Pesaran , M. DiStefano , et al., “Recommendations for Interpreting the Loss of Function PVS1 ACMG/AMP Variant Criterion,” Human Mutation 39, no. 11 (2018): 1517–1524. 30192042 10.1002/humu.23626 PMC6185798

[12] S. Richards , N. Aziz , S. Bale , et al., “Standards and Guidelines for the Interpretation of Sequence Variants: A Joint Consensus Recommendation of the American College of Medical Genetics and Genomics and the Association for Molecular Pathology,” Genetics in Medicine 17, no. 5 (2015): 405–424. 25741868 10.1038/gim.2015.30 PMC4544753

[13] Sequence Variant Interpretation Working Group (SVI WG) , “ClinGen Variant Classification Guidance,” 2025, https://clinicalgenome.org/tools/clingen‐variant‐classification‐guidance/.

[14] M. J. Landrum , J. M. Lee , M. Benson , et al., “ClinVar: Improving Access to Variant Interpretations and Supporting Evidence,” Nucleic Acids Research 46, no. D1 (2017): D1062–D1067. 10.1093/nar/gkx1153 PMC5753237 29165669

[15] P. D. Stenson , M. Mort , E. V. Ball , et al., “The Human Gene Mutation Database: Towards a Comprehensive Repository of Inherited Mutation Data for Medical Research, Genetic Diagnosis and Next‐Generation Sequencing Studies,” Human Genetics 136, no. 6 (2017): 665–677. 28349240 10.1007/s00439-017-1779-6 PMC5429360

[16] ClinVar , “GBE1[gene],” 2025, https://www.ncbi.nlm.nih.gov/clinvar/.

[17] The Genome Aggregation Database (gnomAD) , “gnomADv4.0,” 2025, https://gnomad.broadinstitute.org/news/2023‐11‐gnomad‐v4‐0/.

[18] APBD Research Foundation , “APBD Research Foundation Engages the FDA in Patient Listening Session,” 2021, https://archive.apbdrf.org/wp‐content/uploads/2021/11/FDA‐listening‐Session‐Summary‐FINAL.pdf.

[19] M. A. Hellmann , O. Kakhlon , E. H. Landau , et al., “Frequent Misdiagnosis of Adult Polyglucosan Body Disease,” Journal of Neurology 262, no. 10 (2015): 2346–2351. 26194201 10.1007/s00415-015-7859-4

[20] R. L. Koch , B. T. Kiely , S. J. Choi , et al., “Natural History Study of Hepatic Glycogen Storage Disease Type IV and Comparison to Gbe1ys/Ys Model,” JCI Insight 9, no. 12 (2024): e177722. 38912588 10.1172/jci.insight.177722 PMC11383185

[21] C. Paradas , H. O. Akman , C. Ionete , et al., “Branching Enzyme Deficiency: Expanding the Clinical Spectrum,” JAMA Neurology 71, no. 1 (2014): 41–47. 24248152 10.1001/jamaneurol.2013.4888 PMC6148323

[22] K. Lee , T. Ernst , G. Løhaugen , X. Zhang , and L. Chang , “Neural Correlates of Adaptive Working Memory Training in a Glycogen Storage Disease Type‐IV Patient,” Annals of Clinical and Translational Neurology 4, no. 3 (2017): 217–222. 28275655 10.1002/acn3.394 PMC5338158

[23] I. T. Ferguson , M. Mahon , and W. J. Cumming , “An Adult Case of Andersen's Disease – Type IV Glycogenosis. A Clinical, Histochemical, Ultrastructural and Biochemical Study,” Journal of the Neurological Sciences 60, no. 3 (1983): 337–351. 6579239 10.1016/0022-510x(83)90144-2

[24] M. M. Gayed , P. Sgobbi , W. B. V. R. Pinto , P. S. Kishnani , and R. L. Koch , “Case Report: Expanding the Understanding of the Adult Polyglucosan Body Disease Continuum: Novel Presentations, Diagnostic Pitfalls, and Clinical Pearls,” Frontiers in Genetics 14 (2023): 1282790. 38164512 10.3389/fgene.2023.1282790 PMC10758020

[25] P. V. S. Souza , B. M. L. Badia , I. B. Farias , et al., “GBE1‐Related Disorders: Adult Polyglucosan Body Disease and Its Neuromuscular Phenotypes,” Journal of Inherited Metabolic Disease 44, no. 3 (2021): 534–543. 33141444 10.1002/jimd.12325

[26] APBD Research Foundation , “Foundation Partners With Alex TLC and AGSD‐UK to Host a Special Patient Chat Aimed at Engaging the International Community,” 2025, https://www.apbdrf.org/news‐releases.

[27] gnomAD browser , “Genetic Ancestry,” 2023, https://gnomad.broadinstitute.org/news/2023‐11‐genetic‐ancestry/.

[28] F. Ziemssen , E. Sindern , J. M. Schröder , et al., “Novel Missense Mutations in the Glycogen‐Branching Enzyme Gene in Adult Polyglucosan Body Disease,” Annals of Neurology 47, no. 4 (2000): 536–540. 10762170

[29] I. Colombo , S. Pagliarani , S. Testolin , et al., “Adult Polyglucosan Body Disease: Clinical and Histological Heterogeneity of an Italian Family,” Neuromuscular Disorders 25, no. 5 (2015): 423–428. 25728520 10.1016/j.nmd.2015.01.015

[30] L. Dainese , M. L. Monin , S. Demeret , et al., “Abnormal Glycogen in Astrocytes Is Sufficient to Cause Adult Polyglucosan Body Disease,” Gene 515, no. 2 (2013): 376–379. 23266647 10.1016/j.gene.2012.12.065 PMC7126849

[31] M. A. Franco‐Palacios , M. D. Martin , K. J. Wierenga , et al., “Adult Polyglucosan Body Disease With Reduced Glycogen Branching Enzyme Activity and Heterozygous GBE1 Mutation Mimicking a Low‐Grade Glioma,” International Journal of Clinical and Experimental Pathology 9, no. 3 (2016): 4092–4100.

[32] Y. Harigaya , T. Matsukawa , Y. Fujita , et al., “Novel GBE1 Mutation in a Japanese Family With Adult Polyglucosan Body Disease,” Neurology: Genetics 3, no. 2 (2017): e138. 28265589 10.1212/NXG.0000000000000138 PMC5327677

[33] O. Komure , K. Ichikawa , A. Tsutsumi , K. Hiyama , and A. Fujioka , “Intra‐Axonal Polysaccharide Deposits in the Peripheral Nerve Seen in Adult Polysaccharide Storage Myopathy,” Acta Neuropathologica 65 (1985): 300–304. 3976365 10.1007/BF00687012

[34] T. D. McDonald , P. L. Faust , C. Bruno , S. DiMauro , and J. E. Goldman , “Polyglucosan Body Disease Simulating Amyotrophic Lateral Sclerosis,” Neurology 43 (1993): 785–790. 8469341 10.1212/wnl.43.4.785

[35] A. Sagnelli , M. Savoiardo , C. Marchesi , et al., “Adult Polyglucosan Body Disease in a Patient Originally Diagnosed With Fabry's Disease,” Neuromuscular Disorders 24, no. 3 (2014): 272–276. 24380807 10.1016/j.nmd.2013.11.006

[36] K. Segers , H. Kadhim , C. Colson , R. Duttmann , and G. Glibert , “Adult Polyglucosan Body Disease Masquerading as “ALS With Dementia of the Alzheimer Type”: An Exceptional Phenotype in a Rare Pathology,” Alzheimer Disease and Associated Disorders 26, no. 1 (2012): 96–99. 21572310 10.1097/WAD.0b013e31821cc65d

[37] E. Sindern , F. Ziemssen , T. Ziemssen , et al., “Adult Polyglucosan Body Disease: A Postmortem Correlation Study,” Neurology 61, no. 2 (2003): 263–265. 12874416 10.1212/01.wnl.0000073144.96680.cb

[38] S. Vucic , R. Pamphlett , E. J. Wills , and C. Yiannikas , “Polyglucosan Body Disease Myopathy: An Unusual Presentation,” Muscle & Nerve 35, no. 4 (2007): 536–539. 17221878 10.1002/mus.20720

[39] A. Nagy , A. E. Bley , and F. Eichler , “Canavan Disease,” in GeneReviews(), ed. M. P. Adam (University of Washington, Seattle Copyright 1993‐2022, University of Washington, Seattle. GeneReviews Is a Registered Trademark of the University of Washington, Seattle. All Rights Reserved, 1999).

[40] K. Langer , C. M. Cunniff , and N. Kucine , “Bloom Syndrome,” in GeneReviews(), ed. M. P. Adam (University of Washington, Seattle Copyright 1993–2020, University of Washington, Seattle. GeneReviews is a registered trademark of the University of Washington, Seattle. All rights reserved, 2006).

[41] Biochemical Genetics Laboratory, L., Ontario , “Amish, Mennonite, and Hutterite Genetic Disorders Database,” 2025, https://www.biochemgenetics.ca/plainpeople/view.php.

[42] M. Mulhern , L. Bier , R. N. Alcalay , and M. Balwani , “Patients' Opinions on Genetic Counseling on the Increased Risk of Parkinson Disease Among Gaucher Disease Carriers,” Journal of Genetic Counseling 27, no. 3 (2018): 675–680. 28963610 10.1007/s10897-017-0161-0 PMC5878114

[43] T. G. J. Derks , F. Peeks , F. de Boer , et al., “The Potential of Dietary Treatment in Patients With Glycogen Storage Disease Type IV,” Journal of Inherited Metabolic Disease 44, no. 3 (2021): 693–704. 33332610 10.1002/jimd.12339 PMC8246821

[44] G. d'Orsi , A. Liantonio , P. Imbrici , et al., “Empagliflozin Repurposing for Lafora Disease: A Pilot Clinical Trial and Preclinical Investigation of Novel Therapeutic Targets,” Methods and Protocols 8, no. 3 (2025): 48. 40407475 10.3390/mps8030048 PMC12101192

[45] P. Imbrici , G. d'Orsi , M. Carella , et al., “Sodium‐Glucose Cotransporter‐2 Inhibitors: A Potential Novel Treatment for Lafora Disease?,” Pharmacological Research 199 (2024): 107012. 38036198 10.1016/j.phrs.2023.107012

[46] E. Gumusgoz , S. Kasiri , D. R. Guisso , et al., “AAV‐Mediated Artificial miRNA Reduces Pathogenic Polyglucosan Bodies and Neuroinflammation in Adult Polyglucosan Body and Lafora Disease Mouse Models,” Neurotherapeutics 19, no. 3 (2022): 982–993. 35347645 10.1007/s13311-022-01218-7 PMC9294094

[47] E. Gumusgoz , D. R. Guisso , S. Kasiri , et al., “Targeting Gys1 With AAV‐SaCas9 Decreases Pathogenic Polyglucosan Bodies and Neuroinflammation in Adult Polyglucosan Body and Lafora Disease Mouse Models,” Neurotherapeutics 18, no. 2 (2021): 1414–1425. 33830476 10.1007/s13311-021-01040-7 PMC8423949

[48] B. D. Holt , S. J. Elliott , R. Meyer , et al., “A Novel CD71 Centyrin:Gys1 siRNA Conjugate Reduces Glycogen Synthesis and Glycogen Levels in a Mouse Model of Pompe Disease,” Molecular Therapy 33, no. 1 (2025): 235–248. 39604266 10.1016/j.ymthe.2024.11.033 PMC11764773

[49] J. C. Ullman , R. A. Dick , D. Linzner , et al., “First‐In‐Human Evaluation of Safety, Pharmacokinetics and Muscle Glycogen Lowering of a Novel Glycogen Synthase 1 Inhibitor for the Treatment of Pompe Disease,” Clinical Pharmacology and Therapeutics 116, no. 6 (2024): 1580–1592. 39439155 10.1002/cpt.3470

[50] H. Yi , Q. Zhang , E. D. Brooks , et al., “Systemic Correction of Murine Glycogen Storage Disease Type IV by an AAV‐Mediated Gene Therapy,” Human Gene Therapy 28, no. 3 (2017): 286–294. 27832700 10.1089/hum.2016.099

[51] R. Thomas , E. Miyoshi , H. O. Akman , et al., “Splice‐Modulating Antisense Oligonucleotides Targeting a Pathogenic Intronic Variant in Adult Polyglucosan Body Disease Correct Mis‐Splicing and Restore Enzyme Activity in Patient Cells,” Nucleic Acids Research 53, no. 13 (2025): gkaf658. 40671519 10.1093/nar/gkaf658 PMC12266137

[52] O. Kakhlon , H. Vaknin , K. Mishra , et al., “Alleviation of a Polyglucosan Storage Disorder by Enhancement of Autophagic Glycogen Catabolism,” EMBO Molecular Medicine 13, no. 10 (2021): e14554. 34486811 10.15252/emmm.202114554 PMC8495453

[53] O. Kakhlon , I. Ferreira , L. J. Solmesky , et al., “Guaiacol as a Drug Candidate for Treating Adult Polyglucosan Body Disease,” JCI Insight 3, no. 17 (2018): e99694. 30185673 10.1172/jci.insight.99694 PMC6171812

[54] H. Yi , F. Gao , S. Austin , P. S. Kishnani , and B. Sun , “Alglucosidase Alfa Treatment Alleviates Liver Disease in a Mouse Model of Glycogen Storage Disease Type IV,” Molecular Genetics and Metabolism Reports 9 (2016): 31–33. 27747161 10.1016/j.ymgmr.2016.09.008 PMC5053031

[55] The Orphan Disease Center of the University of Pennsylvania , “Conquer From Within – Treating APBD by Viral Delivery of Cross‐Correction‐Enabled Amylase,” MDBR APBD Awarded Grants 2020, 2026, https://www.orphandiseasecenter.med.upenn.edu/awarded‐grants/discovering‐genetic‐modifiers‐of‐stxbp1‐protein‐stability‐8hfre‐edb54‐yx22l‐3tyg3‐y74z8‐tszwm‐tl8el‐h6gsd?rq=conquer%20from%20within.

[56] P. Sinha , B. Verma , and S. Ganesh , “Trehalose Ameliorates Seizure Susceptibility in Lafora Disease Mouse Models by Suppressing Neuroinflammation and Endoplasmic Reticulum Stress,” Molecular Neurobiology 58, no. 3 (2021): 1088–1101. 33094475 10.1007/s12035-020-02170-3

[57] S. Della Vecchia , A. Ogi , R. Licitra , et al., “Trehalose Treatment in Zebrafish Model of Lafora Disease,” International Journal of Molecular Sciences 23, no. 12 (2022): 6874. 35743315 10.3390/ijms23126874 PMC9224929

[58] H. I. Roessler , N. V. A. M. Knoers , M. van Haelst , and G. van Haaften , “Drug Repurposing for Rare Diseases,” Trends in Pharmacological Sciences 42, no. 4 (2021): 255–267. 33563480 10.1016/j.tips.2021.01.003

[59] W. B. Hannah , M. L. Drumm , K. Nykamp , T. Pramparo , R. D. Steiner , and S. J. Schrodi , “Using Genomic Databases to Determine the Frequency and Population‐Based Heterogeneity of Autosomal Recessive Conditions,” Genetics in Medicine Open 2 (2024): 101881. 39669633 10.1016/j.gimo.2024.101881 PMC11613865

[60] A. Kurtovic‐Kozaric , M. Singer‐Berk , J. Wood , et al., “An Estimation of Global Genetic Prevalence of PLA2G6‐Associated Neurodegeneration,” Orphanet Journal of Rare Diseases 19, no. 1 (2024): 388. 39425167 10.1186/s13023-024-03275-x PMC11489993

[61] gnomAD browser , “What's in gnomAD,” https://gnomad.broadinstitute.org/stats.

[62] All of Us Research Program Genomics Investigators , “Genomic Data in the All of us Research Program,” Nature 627, no. 8003 (2024): 340–346. 38374255 10.1038/s41586-023-06957-x PMC10937371

[63] gnomAD browser , “Federated gnomAD,” https://gnomad.broadinstitute.org/federated.

